# Non-Coding RNA Networks in Pulmonary Hypertension

**DOI:** 10.3389/fgene.2021.703860

**Published:** 2021-11-30

**Authors:** Hongbin Zang, Qiongyu Zhang, Xiaodong Li

**Affiliations:** ^1^ Department of Cardiology, Shengjing Hospital of China Medical University, Shenyang, China; ^2^ Department of Neurology, Shengjing Hospital of China Medical University, Shenyang, China

**Keywords:** pulmonary hypertension, long non-coding RNA, circular RNA, microRNA, network

## Abstract

Non-coding RNAs (ncRNAs) are involved in various cellular processes. There are several ncRNA classes, including microRNAs (miRNAs), long non-coding RNAs (lncRNAs), and circular RNAs (circRNAs). The detailed roles of these molecules in pulmonary hypertension (PH) remain unclear. We systematically collected and reviewed reports describing the functions of ncRNAs (miRNAs, lncRNAs, and circRNAs) in PH through database retrieval and manual literature reading. The characteristics of identified articles, especially the experimental methods, were carefully reviewed. Furthermore, regulatory networks were constructed using ncRNAs and their interacting RNAs or genes. These data were extracted from studies on pulmonary arterial smooth muscle cells, pulmonary artery endothelial cells, and pulmonary artery fibroblasts. We included 14 lncRNAs, 1 circRNA, 74 miRNAs, and 110 mRNAs in the constructed networks. Using these networks, herein, we describe the current knowledge on the role of ncRNAs in PH. Moreover, these networks actively provide an improved understanding of the roles of ncRNAs in PH. The results of this study are crucial for the clinical application of ncRNAs.

## 1 Introduction

Pulmonary hypertension (PH) is a serious disease characterized by progressively increased pulmonary vascular resistance and pulmonary artery pressure; the diagnostic criterion is mean pulmonary artery pressure ≥25 mmHg (Galiè et al., 2016; Weber et al., 2018). The increased pulmonary artery pressure in PH results from changes in the structure and function of the vessel wall, which is induced by abnormal pulmonary cell proliferation, apoptosis, and migration (Bourgeois et al., 2018a). Patients with PH may experience dyspnea, fatigue, syncope, chest pain, and/or edema of the legs and ankles. The causes of PH can be broadly classified as primary and secondary causes. To date, ion channels, vasoactive substances, immune factors, and genetic factors are known to be involved in the pathogenesis of PH (Chelladurai et al., 2016; Veith et al., 2016; Bourgeois et al., 2018b).

Recently, many non-coding RNAs (ncRNAs) have been recognized as important regulators in the development of PH. Most human genes (>95%) do not produce proteins but ncRNA molecules. Among them, microRNAs (miRNAs), long non-coding RNAs (lncRNAs), and circular RNAs (circRNAs) are the most widely studied. MiRNAs are small ncRNAs containing 21–22 nucleotides, which post-transcriptionally regulate gene expression (Wakiyama and Yokoyama. 2014). LncRNAs, which have more than 200 nucleotides, are transcribed from intergenic or intragenic regions. They can bind to proteins, RNA, or DNA to execute regulatory roles (Botti et al., 2017). CircRNAs are a novel class of ncRNAs with a closed loop structure, making them highly stable and capable of interacting with proteins or RNA (Di et al., 2019). NcRNAs have been identified to regulate multiple steps of gene expression. However, because of the large quantity and diverse mechanisms, it is difficult to comprehensively understand the roles of ncRNAs.

NcRNA-based therapeutics have emerged for several diseases, including PH. An effective ncRNA-based strategy demands a thorough understanding of the diverse and context-dependent regulatory relationships of ncRNAs. The regulation of gene expression by ncRNAs is frequently cell specific, suggesting that not only expression level, but also activity or bioavailability contribute to the biofunction of ncRNAs (Correia de Sousa et al., 2019). Thus, in this article, we reviewed the published literature to search for functional miRNAs, lncRNAs, and circRNAs in PH. Next, we constructed networks of validated ncRNAs and their interacting RNAs or genes to investigate the role of ncRNAs in PH.

## 2 Screening of Articles

### 2.1 Criteria for Study Selection

**FIGURE 1:**
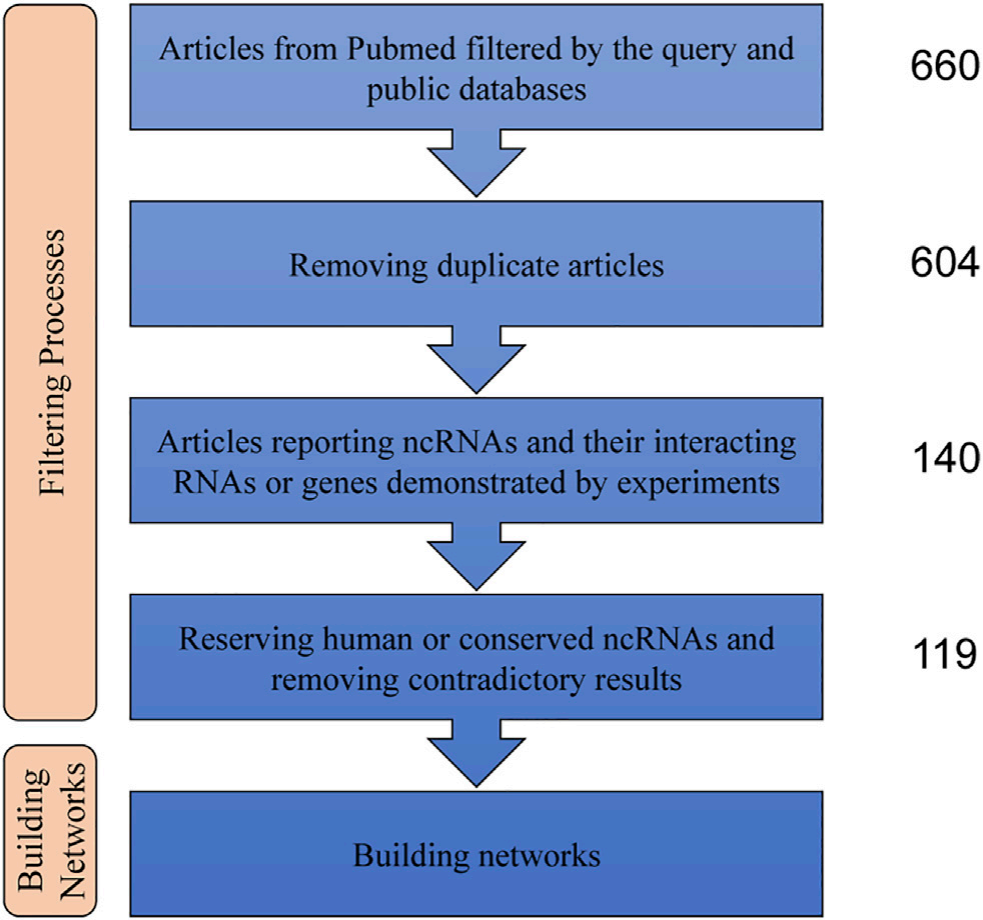
Steps of the data analysis used to build the ncRNA networks. NcRNAs: non-coding RNAs.

A literature search was performed in PubMed with the query listed in Table 1; we identified 602 articles. In addition, we also reviewed other public databases, including the Human microRNA Disease Database v3.2, miRWalk 2.0, and LncRNADisease v2.0, to identify validated functional ncRNAs in PH. Studies were selected when the following criteria were met: 1) the study reported pathogenic roles of miRNAs, lncRNAs, and/or circRNAs in PH; 2) mechanistic studies were performed in pulmonary arterial smooth muscle cells (PASMCs), pulmonary artery endothelial cells (PAECs), and/or pulmonary artery fibroblasts (PAFs); and 3) the relationships between ncRNAs and their interacting RNAs or genes were experimentally identified via luciferase reporter assay, western blot, and/or qPCR. Using these criteria returned 140 qualified articles (Figure 1).

**TABLE 1:** Query for searching articles from PubMed.

Query	Number of articles
(“rna, untranslated"[MeSH Terms] or “non-coding RNA” or “ncRNA” or “noncoding RNA” or “RNA, Long Noncoding"[Mesh] or “long non-coding RNA” or “lncRNA” or “long intergenic non-coding RNA” or “lincRNA” or “RNA, Circular"[Mesh] or “circRNA” or “circular RNA” or “MicroRNAs"[Mesh] or “microRNA” OR “miRNA”) and (“PAH” or “pulmonary hypertension” or “pulmonary artery hypertension")	602

### 2.2 General Characteristics of Qualified Articles

**FIGURE 2:**
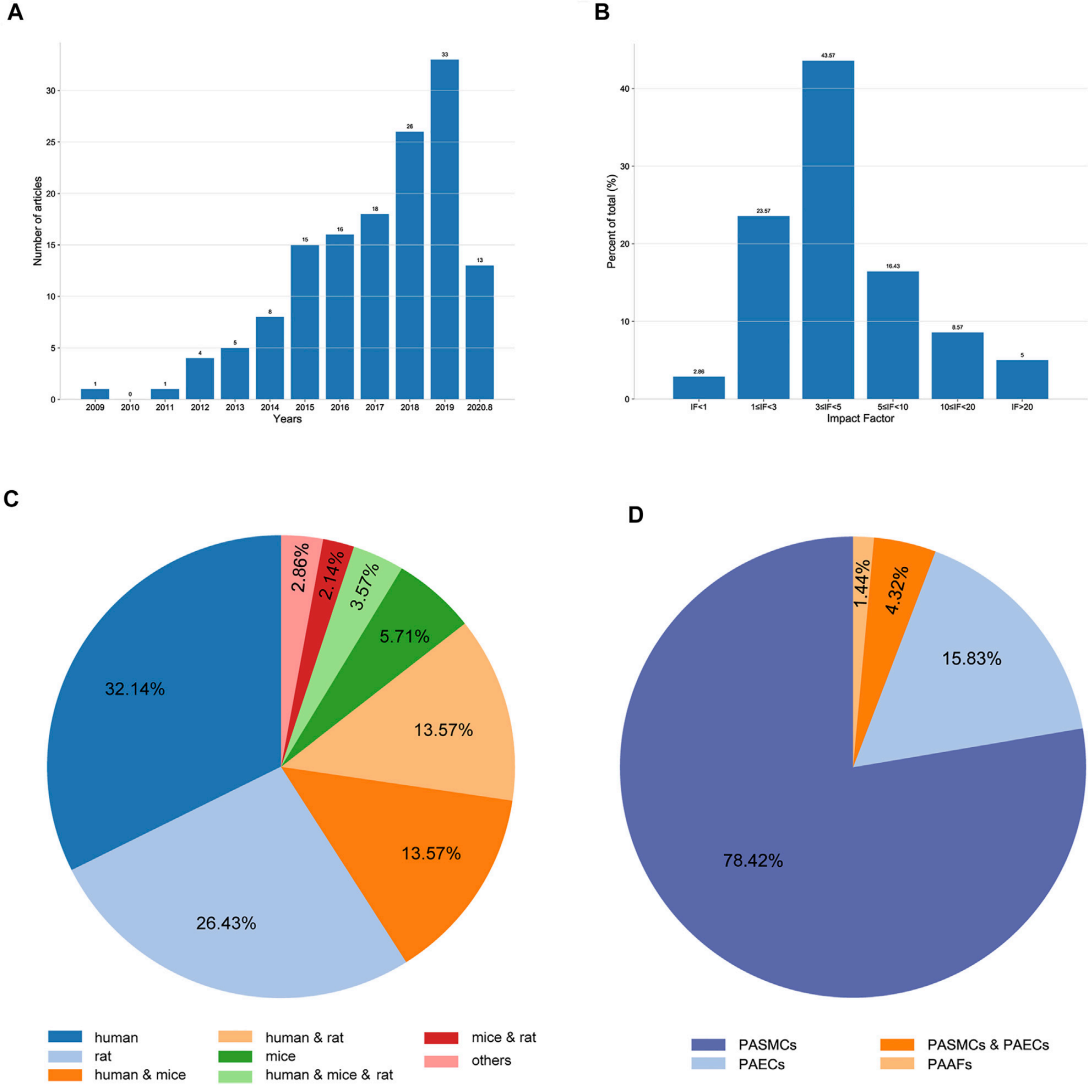
Characteristics of the extracted studies. **(A)** Distribution of the included articles according to the publication year. **(B)** Distribution of the included articles according to the impact factor. **(C)** Proportions of different species studied in the included articles. **(D)** Proportions of different cell types used in the included articles.

When sorted by publication date, we found that the number of eligible articles continuously increased year by year (Figure 2A). The impact factors (IF) of the articles ranged from 0 to 36.13; articles with 3 ≤ IF < 5 accounted for the highest proportion (Figure 2B). Of the 140 qualified articles, 32.14% were studies using human tissues or cells. In studies using experimental animals, rats were the most commonly used, accounting for 26.43% of the total studies (Figure 2C). Moreover, when classified by cell type, 78.42, 15.83, 1.44, and 4.32% of studies were performed in PASMCs, PAECs, PAFs, and both PASMCs and PAECs, respectively (Figure 2D).

## 3 Non-coding RNA Networks for Pulmonary Hypertension

### 3.1 Construction of Non-coding RNA Regulatory Networks

Regulatory networks were constructed using ncRNAs and their interacting RNAs or genes in PASMCs, PAECs, and PAFs. Given ncRNA conservation among species, only human ncRNAs or ncRNAs that were conserved between human and experimental animals were included. If there were contradictory results, the results from higher-impact articles were selected. In addition, some crucial regulatory relationships between protein-coding genes and validated transcription factor–miRNA interactions from TransmiR v2.0 were also described in the networks to present an in-depth explanation on the roles of ncRNAs in PH. The nodes represented interacting molecules, and the edges represented the regulatory connections. Each edge indicated a publication supporting the connection. Square and circular nodes represented ncRNAs and coding RNAs or genes, respectively. Node color was based on the type of molecule (lncRNAs and circRNAs are orange, miRNAs are blue, and coding RNAs or genes are empty). Node sizes represented their degrees (number of edges that directly link to the node). Edges represented the regulatory connections: red edges depicted links indicating repressive action (semicircular arrow heads), and black edges indicated activation (traditional arrow heads). The nodes in this network were involved in cell proliferation, apoptosis, migration, metabolism, endothelial–mesenchymal transition, and extracellular matrix remodeling. The steps used in our approach are shown in Figure 1.

### 3.2 General Characteristics of the Constructed Networks

**FIGURE 3:**
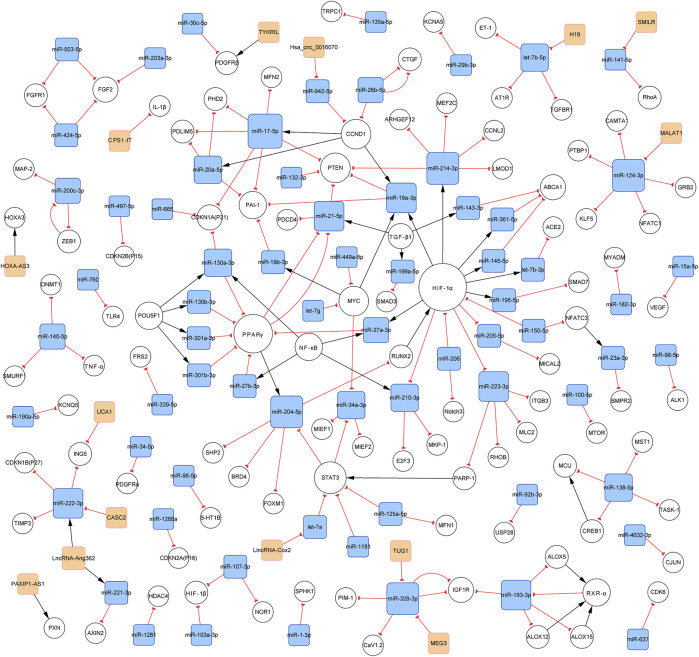
PH-associated network of ncRNAs and their interacting RNAs or genes in PASMCs. The square and circular nodes represent ncRNAs and coding RNAs or genes, respectively. Node color is based on the type of molecule (lncRNAs and circRNAs are orange, miRNAs are blue, and coding RNAs or genes are empty). Node sizes represent the degrees (the number of edges that directly link to the node). Edges represent the regulatory connections, and each edge indicates a publication. When multiple publications describe one interaction, multiple edges connect the same two nodes. Red edges depict links indicating repressive action (semicircular arrow heads), and black edges are those indicating activation (with traditional arrow heads). The nodes in this network are involved in cell proliferation, apoptosis, migration, and metabolism. PH: pulmonary hypertension; PASMCs: pulmonary artery smooth muscle cells; lncRNAs: long non-coding RNAs; circRNA: circular RNA; miRNA: microRNA.

**FIGURE 4:**
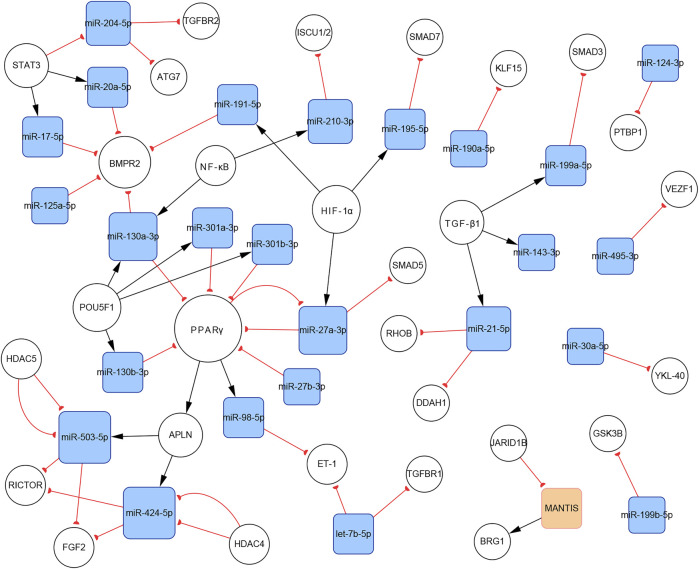
The PH-associated network of ncRNAs and their interacting RNAs or genes in PAECs. The square and circular nodes represent ncRNAs and coding RNAs or genes, respectively. Node color is based on the type of molecule (lncRNAs are orange, miRNAs are blue, and coding RNAs or genes are empty). Node sizes represent the degrees (number of edges that directly link to the node). Edges represent regulatory connections. Each edge indicates a publication. When multiple publications describe one interaction, multiple edges connect the same two nodes. Red edges depict links indicating repressive action (semicircular arrow heads), and black edges represent those indicating activation (traditional arrow heads). The nodes in this network were primarily involved in proliferation, apoptosis resistance, migration, and endothelial–mesenchymal transition. PAECs: pulmonary artery endothelial cells.

**FIGURE 5:**
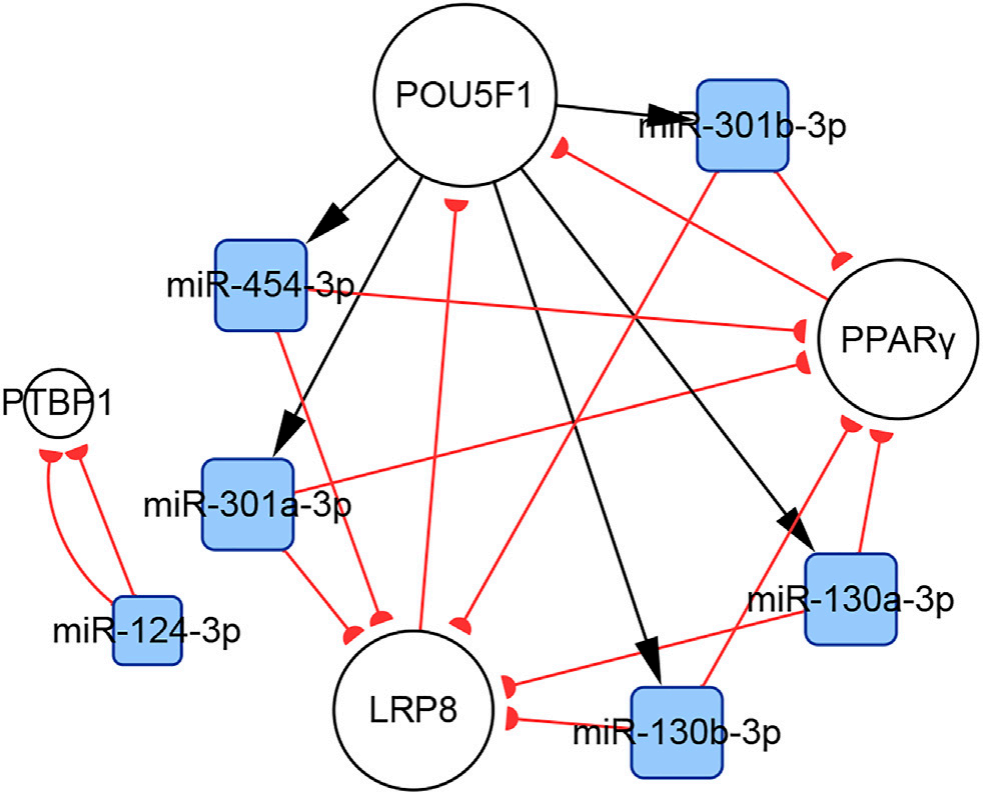
The PH-associated network of ncRNAs and their interacting RNAs or genes in PAFs. The square and circular nodes represent ncRNAs and coding RNAs or genes, respectively. Node color is based on the type of molecule (miRNAs are blue, coding RNAs or genes are empty). Node sizes represent the degrees (number of edges that directly link to the node). Edges represent regulatory connections. Each edge indicates a publication. When multiple publications describe one interaction, multiple edges connect the same two nodes. Red edges depict links indicating repressive action (semicircular arrow heads), and black edges are those indicating activation (traditional arrow heads). The nodes in this network were primarily involved in cell proliferation and extracellular matrix remodeling. PAFs: pulmonary artery fibroblasts.

In total, 140 articles describing 14 lncRNAs, 1 circRNA, 74 miRNAs, and 110 mRNAs, were included in our networks. Considering the unique biological characteristics of different cell types, we constructed networks according to cell type. The network of PASMCs contained 13 lncRNAs, 1 circRNA, 69 miRNAs, and 96 mRNAs. The network of PAECs contained 1 lncRNA, 25 miRNAs, and 29 mRNAs. The network of PAFs contained 6 miRNAs and four mRNAs. The networks are shown in Figures 3–5. Detailed network compositions are listed in Table 2.

**TABLE 2:** List of network interactions.

Upstream molecule	Downstream molecule	Interaction type^a^	PMID	Reference
ALOX12	RXR-α	pos	24963038	Sharma et al. (2014)
ALOX15	RXR-α	pos	24963038	Sharma et al. (2014)
ALOX5	RXR-α	pos	24963038	Sharma et al. (2014)
APLN	miR-424-5p	pos	23263626	Kim et al. (2013)
APLN	miR-503-5p	pos	23263626	Kim et al. (2013)
CASC2	miR-222-3p	neg	32206065	Han et al. (2020)
CCND1	miR-17-5p	pos	18695042	Yu et al. (2008)
CCND1	miR-19a-3p	pos	28090171	Inoue and Fry. (2015)
CCND1	miR-20a-5p	Pos	28090171	Inoue and Fry. (2015)
CPS1-IT	IL-1β	neg	30982984	Zhang et al. (2019b)
CREB1	MCU	pos	27648837	Hong et al. (2017)
H19	let-7b-5p	neg	30547791	Su et al. (2018)
HDAC4	miR-424-5p	neg	29102771	Takagi et al. (2018)
HDAC4	miR-503-5p	neg	29102771	Takagi et al. (2018)
HDAC5	miR-424-5p	neg	29102771	Takagi et al. (2018)
HDAC5	miR-503-5p	neg	29102771	Takagi et al. (2018)
HIF-1α	let-7b-3p	pos	30628484	Zhang H et al. (2019)
HIF-1α	miR-145-5p	pos	25129238	Agrawal et al. (2014)
HIF-1α	miR-191-5p	pos	25119596	Song et al. (2014)
HIF-1α	miR-195-5p	pos	28862358	Zeng et al. (2018)
HIF-1α	miR-19a-3p	pos	31682848	Zhao et al. (2019)
HIF-1α	miR-205-5p	pos	23924028	Gandellini et al. (2014)
HIF-1α	miR-210-3p	neg	22886504	Gou et al. (2012)
HIF-1α	miR-214-3p	pos	24011070	el Azzouzi et al. (2013)
HIF-1α	miR-223-3p	neg	26084306	Meloche et al. (2015a)
HIF-1α	miR-27a-3p	pos	24517586	Camps et al. (2014)
HIF-1α	miR-361-5p	pos	29339076	Zhang Y et al. (2018)
HOXA-AS3	HOXA3	pos	30304383	Zhang R et al. (2019)
Hsa_circ_0016070	miR-942-5p	neg	31593832	Zhou et al. (2019)
JARID1B	MANTIS	neg	2,8351900	Leisegang et al. (2017)
let-7a	STAT3	neg	32803651	Cheng et al. (2020)
let-7b-3p	ACE2	neg	30628484	Zhang Y et al. (2019)
let-7b-5p	AT1R	neg	30547791	Su et al. (2018)
let-7b-5p	ET-1	neg	24978044	Guo et al. (2014)
let-7b-5p	TGFBR1	neg	24978044	Guo et al. (2014)
let-7g	MYC	neg	27889560	Zhang W.-F et al. (2017)
LincRNA-Cox2	let-7a	neg	32803651	Cheng et al. (2020)
LncRNA-Ang362	miR-221-3p	pos	31313741	Wang et al. (2020)
LncRNA-Ang362	miR-222-3p	pos	31313741	Wang et al. (2020)
LRP8	POU5F1	neg	26565914	Bertero et al. (2015)
MALAT1	miR-124-3p	neg	31257528	Wang S et al. (2019)
MANTIS	BRG1	pos	2,8351900	Leisegang et al. (2017)
MEG3	miR-328-3p	neg	31477557	Xing X.-Q et al. (2019)
miR-100-5p	MTOR	neg	26409044	Wang et al. (2015)
miR-103a-3p	HIF-1β	neg	26827991	Deng et al. (2016)
miR-107-3p	HIF-1β	neg	26827991	Deng et al. (2016)
miR-107-3p	NOR1	neg	31933977	Chen et al. (2019)
miR-1181	STAT3	neg	30211651	Qian et al. (2018)
miR-124-3p	CAMTA1	neg	23853098	Kang K et al. (2013)
miR-124-3p	GRB2	neg	28496318	Li Y et al. (2017)
miR-124-3p	KLF5	neg	31257528	Wang D et al. (2019)
miR-124-3p	NFATC1	neg	23853098	Kang B.-Y et al. (2013)
miR-124-3p	PTBP1	neg	23853098	Kang K et al. (2013)
miR-124-3p	PTBP1	neg	24122720	Wang et al. (2014)
miR-124-3p	PTBP1	neg	2,8971999	Caruso et al. (2017)
miR-124-3p	PTBP1	neg	2,8972001	Zhang H et al. (2017)
miR-125a-5p	BMPR2	neg	25854878	Huber et al. (2015)
miR-125a-5p	MFN1	neg	28593577	Ma et al. (2017)
miR-125a-5p	STAT3	neg	29700287	Cai et al. (2018)
miR-1268a	CDKN2A(P16)	neg	31370272	Lee and Kang. (2019)
miR-1281	HDAC4	neg	29514810	Li et al. (2018)
miR-130a-3p	BMPR2	neg	28755990	Li L et al. (2017)
miR-130a-3p	CDKN1A(P21)	neg	25681685	Brock et al. (2015)
miR-130a-3p	LRP8	neg	26565914	Bertero et al. (2015)
miR-130a-3p	PPARγ	neg	24960162	Bertero et al. (2014)
miR-130a-3p	PPARγ	neg	26565914	Bertero et al. (2015)
miR-130b-3p	LRP8	neg	26565914	Bertero et al. (2015)
miR-130b-3p	PPARγ	neg	24960162	Bertero et al. (2014)
miR-130b-3p	PPARγ	neg	26565914	Bertero et al. (2015)
miR-132-3p	PTEN	neg	30896881	Zeng et al. (2019)
miR-135a-5p	TRPC1	neg	30038339	Liu A et al. (2019)
miR-138-5p	CREB1	neg	27648837	Hong et al. (2017)
miR-138-5p	MCU	neg	27648837	Hong et al. (2017)
miR-138-5p	MST1	neg	23485012	Li et al. (2013)
miR-138-5p	TASK-1	neg	29257242	Liu G et al. (2018)
miR-1-3p	SPHK1	neg	29167124	Sysol et al. (2018)
miR-140-5p	DNMT1	neg	27021683	Zhang and Xu. (2016)
miR-140-5p	SMURF1	neg	27214554	Rothman et al. (2016)
miR-140-5p	TNF-α	neg	30367500	Zhu et al. (2019b)
miR-143-3p	ABCA1	neg	30195228	Yue et al. (2018)
miR-141-5p	RHOA	neg	32559140	Lei et al. (2020)
miR-145-5p	ABCA1	neg	30195228	Yue et al. (2018)
miR-150-5p	HIF-1α	neg	28715868	Chen M et al. (2017)
miR-150-5p	NFATC3	neg	30551428	Li et al. (2019)
miR-15a-5p	VEGF	neg	31894295	Zhang et al. (2020)
miR-17-5p	BMPR2	neg	19390056	Brock et al. (2009)
miR-17-5p	CDKN1A(P21)	neg	30305109	Liu J. J et al. (2018)
miR-17-5p	MFN2	neg	27640178	Lu et al. (2016)
miR-17-5p	PAI-1	neg	29644896	Chen K.-H et al. (2018)
miR-17-5p	PDLIM5	neg	25647182	Chen et al. (2015)
miR-17-5p	PHD2	neg	27919930	Chen et al. (2016)
miR-17-5p	PTEN	neg	30305109	Liu G et al. (2018)
miR-182-3p	MYADM	neg	32373233	Sun et al. (2020)
miR-190a-5p	KCNQ5	neg	24446351	Li et al. (2014)
miR-190a-5p	KLF15	neg	30538440	Jiang et al. (2018)
miR-191-5p	BMPR2	neg	31119161	Zhang Z et al. (2019)
miR-193-3p	ALOX12	neg	24963038	Sharma et al. (2014)
miR-193-3p	ALOX15	neg	24963038	Sharma et al. (2014)
miR-193-3p	ALOX5	neg	24963038	Sharma et al. (2014)
miR-193-3p	IGF1R	neg	24963038	Sharma et al. (2014)
miR-195-5p	SMAD7	neg	28862358	Zeng et al. (2018)
miR-199a-5p	SMAD3	neg	27038547	Liu H et al. (2016)
miR-199b-5p	GSK3B	neg	27188753	Wu et al. (2016)
miR-19a-3p	PAI-1	neg	29644896	Chen T et al. (2018)
miR-19a-3p	PTEN	neg	31682848	Zhao et al. (2019)
miR-19b-3p	PAI-1	neg	29644896	Chen K.-H et al. (2018)
miR-200c-3p	MAP2	neg	29044995	Yuan et al. (2017)
miR-200c-3p	ZEB1	neg	29044995	Yuan et al. (2017)
miR-203a-3p	FGF2	neg	30575929	Wang et al. (2018)
miR-204-5p	ATG7	neg	31542480	Liu H.-M et al. (2019)
miR-204-5p	BRD4	neg	26224795	Meloche et al. (2015a)
miR-204-5p	FOXM1	neg	29290032	Bourgeois et al. (2018b)
miR-204-5p	RUNX2	neg	27149112	Ruffenach et al. (2016)
miR-204-5p	SHP2	neg	21321078	Courboulin et al. (2011)
miR-204-5p	TGFBR2	neg	29196166	Yu et al. (2018)
miR-205-5p	MICAL2	neg	30853343	Tao et al. (2019)
miR-206	Notch3	neg	23071643	Jalali et al. (2012)
miR-206	HIF-1α	neg	23628900	Yue et al. (2013)
miR-20a-5p	BMPR2	neg	19390056	Brock et al. (2009)
miR-20a-5p	PAI-1	neg	29644896	Chen T et al. (2018)
miR-20a-5p	PDLIM5	neg	25647182	Chen et al. (2015)
miR-20a-5p	PHD2	neg	27919930	Chen et al. (2016)
miR-210-3p	E2F3	neg	22886504	Gou et al. (2012)
miR-210-3p	ISCU1/2	neg	25825391	White et al. (2015)
miR-210-3p	MKP-1	neg	25044272	Jin et al. (2015)
miR-214-3p	ARHGEF12	neg	31373336	Xing Y et al. (2019)
miR-214-3p	CCNL2	neg	27381447	Liu Y et al. (2016)
miR-214-3p	LMOD1	neg	27144530	Sahoo et al. (2016)
miR-214-3p	MEF2C	neg	27144530	Sahoo et al. (2016)
miR-214-3p	PTEN	neg	28684904	Liu et al. (2017)
miR-21-5p	DDAH1	neg	24895913	Iannone et al. (2014)
miR-21-5p	PDCD4	neg	28522568	Green et al. (2017)
miR-21-5p	PTEN	neg	26208095	Green et al. (2015)
miR-21-5p	RHOB	neg	22371328	Parikh et al. (2012)
miR-221-3p	AXIN2	neg	28694128	Nie et al. (2019)
miR-222-3p	ING5	neg	32206065	Han et al. (2020)
miR-222-3p	CDKN1B(P27)	neg	28854428	Xu et al. (2017)
miR-222-3p	TIMP3	neg	28854428	Xu et al. (2017)
miR-223-3p	ITGB3	neg	30507047	Liu et al. (2019a)
miR-223-3p	MLC2	neg	27121304	Zeng et al. (2016)
miR-223-3p	PARP1	neg	26084306	Meloche et al. (2015b)
miR-223-3p	RHOB	neg	27121304	Zeng et al. (2016)
miR-23a-3p	BMPR2	neg	29864909	Zhang X et al. (2018)
miR-26b-5p	CCND1	neg	2,7322082	Wang P et al. (2016)
miR-26b-5p	CTGF	neg	2,7322082	Wang R et al. (2016)
miR-26b-5p	CTGF	neg	28816418	Zhou et al. (2018)
miR-27a-3p	PPARγ	neg	24244514	Kang B.-Y et al. (2013)
miR-27a-3p	PPARγ	neg	28484848	Xie et al. (2017)
miR-27a-3p	SMAD5	neg	31004656	Liu et al. (2019b)
miR-27b-3p	PPARγ	neg	25795136	Bi et al. (2015)
miR-27b-3p	PPARγ	neg	28484848	Xie et al. (2017)
miR-29b-3p	KCNA5	neg	31553627	Babicheva et al. (2020)
miR-301a-3p	LRP8	neg	26565914	Bertero et al. (2015)
miR-301a-3p	PPARγ	neg	24960162	Bertero et al. (2014)
miR-301a-3p	PPARγ	neg	26565914	Bertero et al. (2015)
miR-301b-3p	LRP8	neg	26565914	Bertero et al. (2015)
miR-301b-3p	PPARγ	neg	24960162	Bertero et al. (2014)
miR-301b-3p	PPARγ	neg	26565914	Bertero et al. (2015)
miR-30a-5p	YKL-40	neg	31115541	Tan et al. (2019)
miR-30c-5p	PDGFRβ	neg	25882492	Xing et al. (2015)
miR-328-3p	CaV1.2	neg	22392900	Guo et al. (2012)
miR-328-3p	IGF1R	neg	22392900	Guo et al. (2012)
miR-328-3p	IGF1R	neg	31477557	Xing X.-Q et al. (2019)
miR-328-3p	PIM-1	neg	27448984	Qian et al. (2016)
miR-339-5p	FRS2	neg	28947594	Chen J et al. (2017)
miR-34-5p	PDGFRα	neg	27302634	Wang P et al. (2016)
miR-34a-3p	MIEF1	neg	29431643	Chen K.-H et al. (2018)
miR-34a-3p	MIEF2	neg	29431643	Chen T et al. (2018)
miR-361-5p	ABCA1	neg	29339076	Zhang Y et al. (2018)
miR-424-5p	FGF2	neg	23263626	Kim et al. (2013)
miR-424-5p	FGF2	neg	24960162	Bertero et al. (2014)
miR-424-5p	FGFR1	neg	23263626	Kim et al. (2013)
miR-424-5p	RICTOR	neg	29102771	Takagi et al. (2018)
miR-449a-5p	MYC	neg	30715622	Zhang et al. (2019a)
miR-454-3p	LRP8	neg	26565914	Bertero et al. (2015)
miR-454-3p	PPARγ	neg	26565914	Bertero et al. (2015)
miR-4632-3p	CJUN	neg	28701355	Qian et al. (2017)
miR-495-3p	VEZF1	neg	31030195	Fu et al. (2019)
miR-497-5p	CDKN2B(P15)	neg	31370272	Lee and Kang. (2019)
miR-503-5p	FGF2	neg	23263626	Kim et al. (2013)
miR-503-5p	FGF2	neg	24960162	Bertero et al. (2014)
miR-503-5p	FGFR1	neg	23263626	Kim et al. (2013)
miR-503-5p	RICTOR	neg	29102771	Takagi et al. (2018)
miR-637	CDK6	neg	27794186	Sang et al. (2016)
miR-665	CDKN1A(P21)	neg	31370272	Lee and Kang. (2019)
miR-760	TLR4	neg	30226538	Yang et al. (2018)
miR-92b-3p	USP28	neg	30149918	Hao et al. (2018)
miR-942-5p	CCND1	neg	31593832	Zhou et al. (2019)
miR-96-5p	5-HT1B	neg	25871906	Wallace et al. (2015)
miR-98-5p	ALK1	neg	31322216	Li et al. (2019)
miR-98-5p	ET-1	neg	26098770	Kang et al. (2016)
MYC	miR-19a-3p	pos	17943719	Schulte et al. (2008)
MYC	miR-19b-3p	pos	17943719	Schulte et al. (2008)
MYC	miR-34a-3p	neg	18066065	Chang et al. (2008)
NFATC3	miR-23a-3p	pos	19574461	Lin et al. (2009)
NF-κB	miR-130a-3p	pos	28755990	Li Q et al. (2017)
NF-κB	miR-210-3p	pos	25341039	Liu et al. (2014)
NF-κB	miR-27a-3p	pos	28484848	Xie et al. (2017)
NF-κB	miR-27b-3p	pos	28484848	Xie et al. (2017)
PARP-1	STAT3	pos	24270264	Meloche et al. (2014)
PAXIP1-AS1	PXN	pos	30450722	Jandl et al. (2019)
POU5F1	miR-130a-3p	pos	24960162	Bertero et al. (2014)
POU5F1	miR-130a-3p	pos	26565914	Bertero et al. (2015)
POU5F1	miR-130b-3p	pos	24960162	Bertero et al. (2014)
POU5F1	miR-130b-3p	pos	26565914	Bertero et al. (2015)
POU5F1	miR-301a-3p	pos	24960162	Bertero et al. (2014)
POU5F1	miR-301a-3p	pos	26565914	Bertero et al. (2015)
POU5F1	miR-301b-3p	pos	24960162	Bertero et al. (2014)
POU5F1	miR-301b-3p	pos	26565914	Bertero et al. (2015)
POU5F1	miR-454-3p	pos	26565914	Bertero et al. (2015)
PPARγ	APLN	pos	24960162	Bertero et al. (2014)
PPARγ	miR-204-5p	pos	24960162	Bertero et al. (2014)
PPARγ	miR-21-5p	neg	26208095	Green et al. (2015)
PPARγ	miR-21-5p	neg	28522568	Green et al. (2017)
PPARγ	miR-27a-3p	neg	24244514	Kang K et al. (2013)
PPARγ	miR-98-5p	pos	26098770	Kang et al. (2016)
PPARγ	POU5F1	neg	26565914	Bertero et al. (2015)
RUNX2	HIF-1α	pos	27149112	Ruffenach et al. (2016)
RXR-α	miR-193-3p	neg	24963038	Sharma et al. (2014)
SMILR	miR-141-5p	neg	32559140	Lei et al. (2020)
STAT3	miR-17-5p	pos	19390056	Brock et al. (2009)
STAT3	miR-204-5p	neg	23975026	Xu et al. (2013)
STAT3	miR-20a-5p	pos	19390056	Brock et al. (2009)
STAT3	miR-34a-3p	neg	24642471	Rokavec et al. (2014)
TGF-β1	miR-143-3p	pos	2,6311719	Deng et al. (2015)
TGF-β1	miR-199a-5p	pos	20705240	Davis et al. (2010)
TGF-β1	miR-21-5p	pos	20705240	Davis et al. (2010)
TUG1	miR-328-3p	neg	31679623	Wang D et al. (2019)
TYKRIL	PDGFRβ	pos	32634060	Zehendner et al. (2020)
UCA1	ING5	neg	30353369	Zhu et al. (2019a)
ZEB1	miR-200c-3p	neg	18829540	Bracken et al. (2008)

a pos: positive interaction, neg: negative interaction.

### 3.3 Functional Enrichment Analysis

**FIGURE 6:**
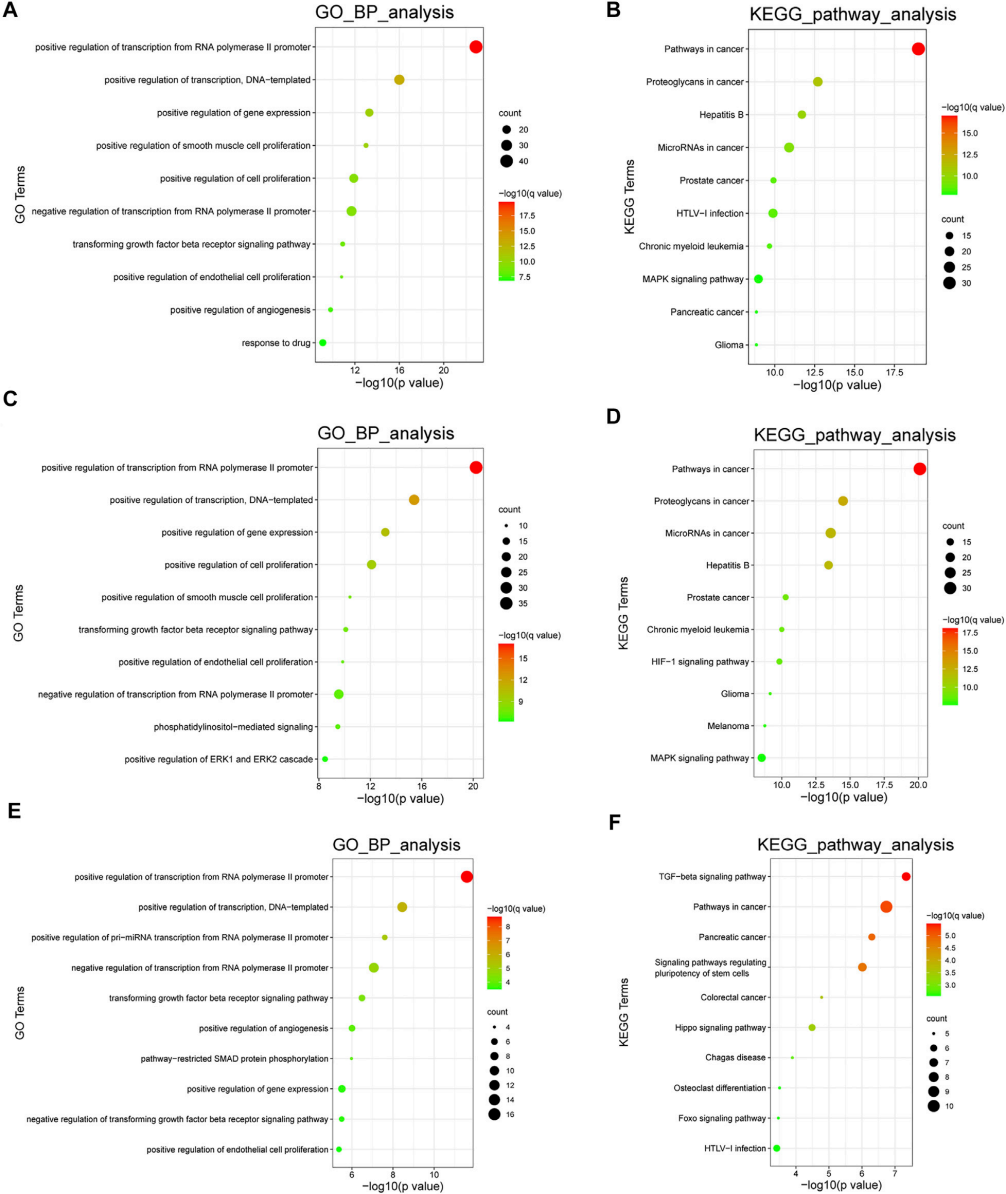
Functional analyses of the ncRNAs. **(A,B)**. The top 10 enriched GO biological process terms and KEGG pathways in all cell types. **(C,D)** The top 10 enriched GO biological process terms and KEGG pathways in PASMCs. **(E,F)** The top 10 enriched GO biological process terms and KEGG pathways in PAECs. Node sizes indicate the number of genes enriched in functional clusters. Node colors are related to q values. GO: gene ontology; KEGG: Kyoto Encyclopedia of Genes and Genomes.

We performed gene ontology (GO) biological process term analyses and Kyoto Encyclopedia of Genes and Genomes (KEGG) pathway analyses using the database for Annotation, Visualization, and Integration Discovery (DAVID). The calculation process was dependent on a hypergeometric test, using a statistical significance threshold of *p* < 0.05 with a false discovery rate (FDR) correction. These analyses provided a general overview of the biological roles of the included ncRNAs. In addition, we performed cell type-specific functional enrichment analyses. However, owing to the lack of sufficient molecules, the enrichment analysis in PAFs could not be conducted. GO analysis and KEGG pathway enrichment in PASMCs and PAECs revealed several PH-associated terms, such as positive regulation of smooth muscle cell proliferation, positive regulation of endothelial cell proliferation, HIF-1 signaling pathway, and MAPK signaling pathway. The top 10 enriched GO biological process terms and KEGG pathways are shown in Figure 6.

### 3.4 Key Non-coding RNA Subnetworks

We built three networks according to the cell types. Here, we discuss several important subnetworks, along with their components and interactions, to improve understanding of the roles of ncRNAs in PH. Subnetworks with more than five nodes were regarded as key subnetworks.

#### 3.4.1 The Hsa_circ_0016070/miR-942-5p/CCND1 Subnetwork

**FIGURE 7:**
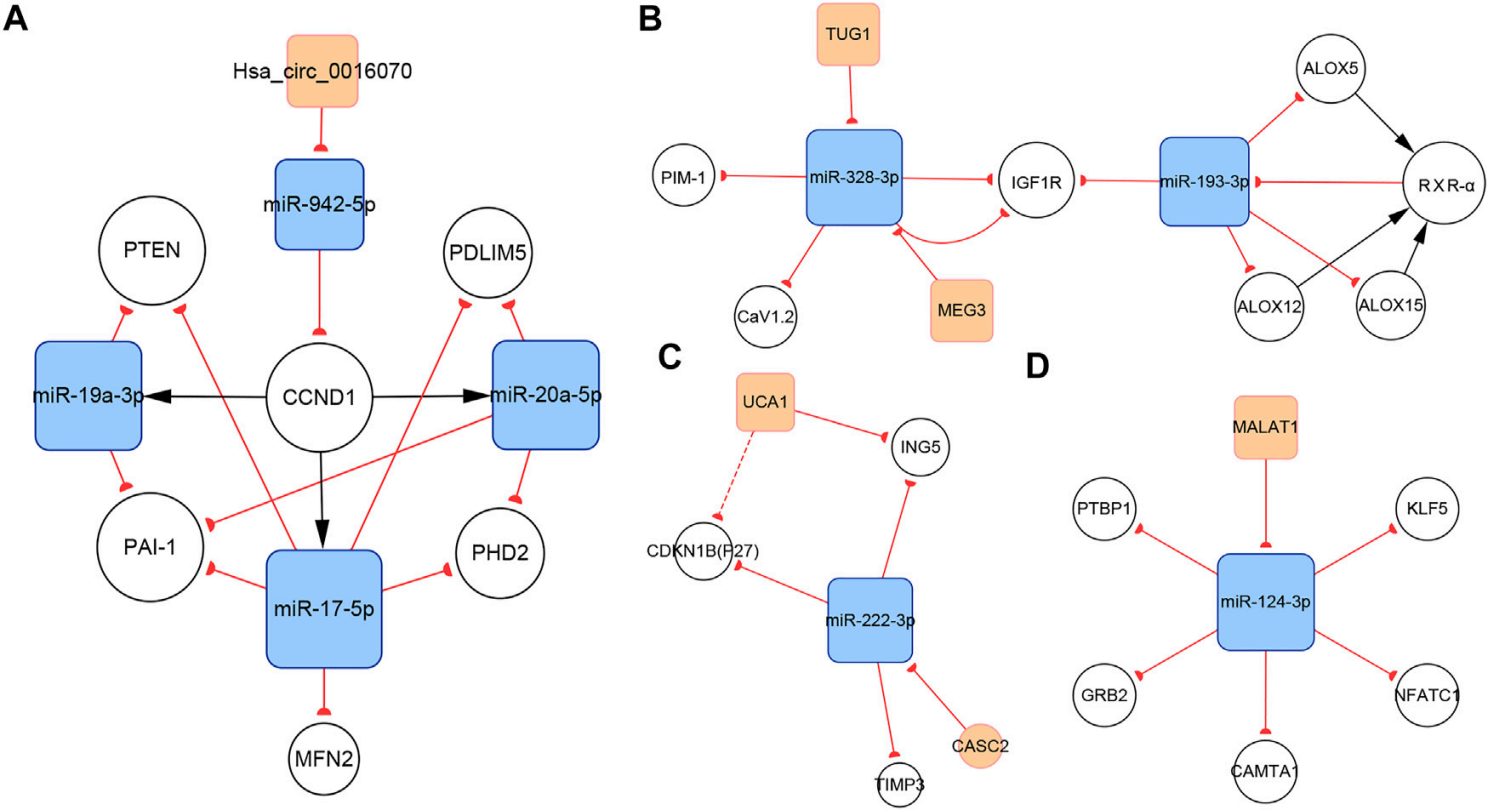
Several key ncRNA subnetworks. **(A)** The hsa_circ_0016070/miR-942-5p/CCND1 subnetwork. **(B)** The TUG1/MEG3/miR-328-3p/miR-193-3p subnetwork. **(C)** The CASC2/UCA1/miR-222-3p subnetwork. **(D)** The MALAT1/miR-124-3p subnetwork.

CircRNAs are associated with various cardiovascular diseases. Hsa_circ_0016070 was the only circRNA included in our networks. This circRNA is located at chr1: 203595914-203702528, strand: +, promotes cell proliferation by mediating cell cycle progression, and is increased in PH patients (Zhou et al., 2019). CCND1 is an important regulator of the cell cycle. It interacts with cyclin-dependent kinase 4 (CDK4) to form the cyclin D1–CKD4 complex, which then inactivates retinoblastoma (Rb) protein and induces G0 progression to S phase (Matsushime et al., 1991). The subnetwork showed that hsa_circ_0016070 overexpression induced CCND1 expression by buffering miR-942-5p (Zhou et al., 2019). In addition, according to our network, CCND1 could induce the expression of miR-17-5p, miR-19a-3p, and miR-20a-5p, subsequently regulating the biological activities of PASMCs (Figure 7A). Given the considerable number of identified circRNAs, there should be other PH-related circRNAs. A microarray expression profile in thromboembolic pulmonary hypertension patients indicated that hsa_circ_0002062 and hsa_circ_0022342 might be the key circRNAs for the development of chronic thromboembolic pulmonary hypertension (Miao et al., 2017). However, this finding has not been verified by more reliable experimental methods.

#### 3.4.2 The TUG1/MEG3/miR-328-3p/miR-193-3p Subnetwork

Regulatory relationships are indicated by the connection lines in the subnetwork. According to this subnetwork (Figure 7B), both TUG1 and MEG3 can function as competing endogenous RNAs (ceRNAs) that sequester miR-328-3p. In the original studies, the TUG1/miR-328-3p and MEG3/miR-328-3p axes were identified (Wang D et al., 2019; Xing X.-Q et al., 2019). IGF1 is reported to inhibit PASMCs apoptosis and activate elastin in PASMCs. Thus, upregulating IGF1R via the TUG1/miR-328-3p and MEG3/miR-328-3p axes can induce PH by amplifying the pathogenic role of IGF1 (Wang S et al., 2019; Xing Y et al., 2019). Calcium voltage-gated channel subunit alpha1 C (CaV1.2), which contributes to vasoconstriction, is also a target gene of miR-328-3p in PASMCs (Guo et al., 2012), indicating that the TUG1/miR-328-3p and MEG3/miR-328-3p axes are involved in regulating pulmonary artery contraction and dilation. In addition, miR-328-3p can inhibit PASMC proliferation by targeting PIM-1 (Qian et al., 2016). Available data show that miR-193-3p has a shared target gene, IGF1R, with miR-328-3p, but no strong regulatory connection with miR-328-3p or TUG1 or MEG3. Thus, downregulation of miR-193-3p contributes to IGF1R overexpression as well. In addition, miR-193-3p is capable of negatively regulating multiple lipoxygenases, including ALOX5, ALOX12, and ALOX15. These lipoxygenases cause abnormal lipid metabolism, which not only directly accelerates the development of PH, but also induces the increasement of RXR-α. Moreover, miR-193-3p can be downregulated by RXR-α, which directly binds to the miR-193 promoter. Therefore, a feedback loop, which dramatically enhances abnormal miR-193-3p expression forms (Sharma et al., 2014).

#### 3.4.3 The CASC2/UCA1/miR-222-3p Subnetwork

LncRNA CASC2 is downregulated in hypoxia-induced PASMCs. As a ceRNA of miR-222-3p, CASC2 reduces the expression of ING5, which is a target gene of miR-222-3p, ultimately promoting PASMC proliferation and migration (Han et al., 2020). P27 and TIMP3 are two additional target genes of miR-222-3p (Xu et al., 2017). P27, a member of the Cip/Kip family of cyclin-dependent kinase inhibitors, negatively regulates cell proliferation (Toyoshima and Hunter, 1994). Meanwhile, TIMP3 is a member of the TIMP family, which regulates cell proliferation, apoptosis, and migration via both MMP-dependent or MMP-independent pathways (Zhou et al., 2015). The present subnetwork links CACS2 to P27 and TIMP3 via miR-222-3p, further elaborating the mechanisms of PH (Figure 7C).

UCA1 is the other lncRNA in this subnetwork and is highly expressed in hypoxia-induced PASMCs. Studies indicate that UCA1 does not interact with miR-222-3p, but directly inhibits ING5 by competing with ING5 mRNA for hnRNP I, which binds to ING5 mRNA and enhances its translation. Thus, UCA1 overexpression results in the downregulation of ING5 mRNA expression (Zhu T.-T. et al., 2019). The same regulatory pattern has been found between UCA1 and P27 in breast tumor studies (Huang et al., 2014). This interaction may also work in PH and partly contributes to P27 downregulation (Figure 7C).

#### 3.4.4 The MALAT1/miR-124-3p Subnetwork

LncRNA MALAT1, located at 11q13, is an 8.5-kb molecule that was identified by Ji et al. in a cancer study (Ji et al., 2003). Emerging evidence indicates that MALAT1 plays important roles in various diseases, including PH. Wang et al. reported that MALAT1 is highly expressed in pulmonary artery tissues and PASMCs from patients with PH. MALAT1 controls PASMC proliferation and migration by binding to miR-124-3p, which directly targets KLF5 (Wang D et al., 2019). Kang et al. showed that miR-124-3p also targets three regulators of the NFAT pathway, including NFATc1, CAMTA1, and PTBP1 (Kang B.-Y et al., 2013). The downregulation of miR-124-3p induces PASMC proliferation and reverses the differentiated PASMC phenotype by activating the NFAT pathway. In addition to its role in PASMCs, miR-124-3p also regulates the biological behaviors of PAH endothelial cells (PAH ECs) and PAFs. Studies have confirmed the role of the miR-124-3p/PTBP1 axis in PAH ECs and PAFs (Caruso et al., 2017; Wang et al., 2014; Zhang H et al., 2017). Downregulating miR-124-3p activates PTBP1 expression, which promotes aerobic glycolysis by increasing the PKM2/PKM1 ratio, subsequently inducing PAH EC and PAF proliferation (Anastasiou et al., 2012). Li et al. reported another target of miR-124-3p, GRB2, which enhanced the proliferation of multiple human cells (Li L et al., 2017; Figure 7D).

#### 3.4.5 Subnetworks of the miR-130/301 Family

There are complicated relationships between the miR-130/301 family and other functional molecules associated with the pathogenesis of PH. In the present study, we found that subnetworks of the miR-130/301 family were involved in multiple biological behaviors, such as proliferation, apoptosis, and migration in PASMCs, PAECs, and PAFs. In addition, these subnetworks also mediated the crosstalk of these pulmonary artery cells.

**FIGURE 8:**
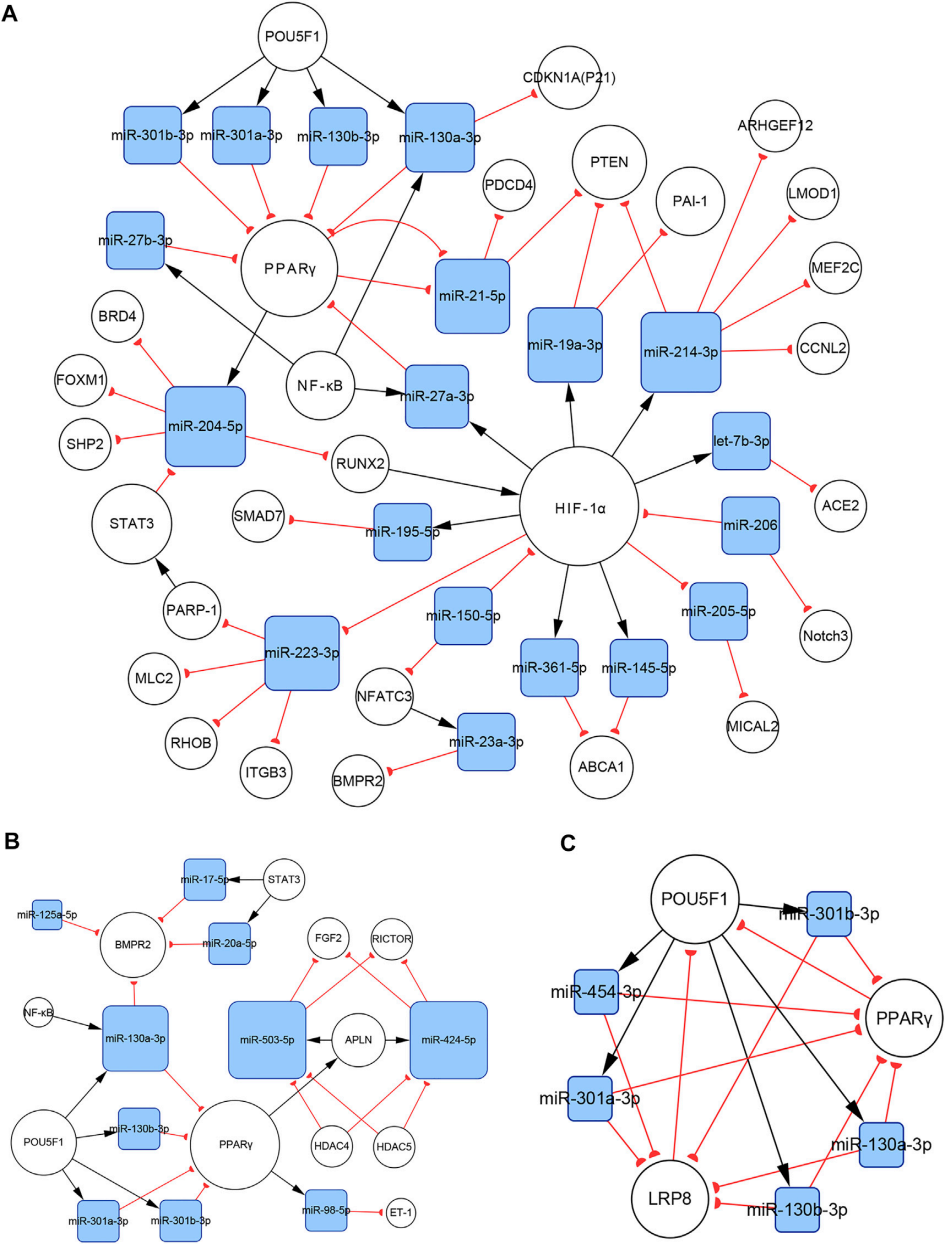
The miR-130/301 family subnetworks in **(A)** PASMCs, **(B)** PAECs, and **(C)** PAFs. This miRNA family was involved in multiple processes, such as cell proliferation, apoptosis, migration, endothelial contraction, and matrix remodeling.

In PASMCs, the miR-130/301 family is involved in many regulatory axes. Among them, the POU5F1/miR-130/301 family/PPARγ axis, which regulates the expression of miR-204-5p and miR-21-5p, is the most explicitly elaborated axis. According to our studies, the identified target genes of the two miRNAs in PASMCs include BRD4, FOXM1, PSCD4, PTEN, RUNX2, and SHP2, which control cell proliferation, apoptosis, differentiation, and mitochondrial function (Courboulin et al., 2011; Meloche et al., 2015a; Green et al., 2015, 2017; Ruffenach et al., 2016; Liu et al., 2017; Bourgeois et al., 2018a). In addition to the miR-130/301 family, miR-27a/b-3p, which is regulated by NF-κB (Xie et al., 2017), can also act as an upstream controller of PPARγ in PASMCs. Interestingly, the subnetwork analysis indicates that the miR-130/301 family indirectly promotes HIF-1α expression by sustaining the RUNX2 level (Ruffenach et al., 2016). Conversely, HIF-1α induces the expression of miR-27a-3p, which depresses the level of PPARγ (Camps et al., 2014). Thus, a feedback loop with PPARγ and HIF-1α forms. This loop leads to a persistent pathological status. Moreover, as a crucial pathogenic molecule for PH, HIF-1α can function through several miRNAs, including miR-145-5p, miR-19a-3p, miR-195-5p, miR-210-3p, miR-223-3p, and miR-361-5p, to regulate the expression of downstream proteins, eventually causing abnormal cellular behaviors (Agrawal et al., 2014; Gou et al., 2012; Meloche et al., 2015b; Zeng et al., 2018; Zhang X et al., 2018, Zhang H et al., 2019; Zhao et al., 2019; Figure 8A).

In PAECs, the miR-130/301 family also plays an important role. The POU5F1/miR-130/301 family/PPARγ axis indirectly regulates the expression of ET-1 and FGF2 via miR-98-5p and miR-424/503-5p, respectively (Kim et al., 2013; Zhang Y et al., 2018). The roles of ET-1 and FGF2 in PH are well established. ET-1 is synthesized primarily in endothelial cells and mediates pulmonary artery cell proliferation, migration, and constriction through two distinct G protein-coupled receptors: ETA and ETB (Clozel, 2016). Previous studies suggest that excessive FGF2 expression promotes PAEC proliferation by activating ERK1/2 and inhibits apoptosis by inducing BCL2 and BCL-xL activity (Tu et al., 2011). Furthermore, miR-130a-3p controls the level of BMPR2, which triggers idiopathic pulmonary artery hypertension (IPAH) and is involved in the development of other types of PH (Li Q et al., 2017). Considering that miRNAs from the same family have a homologous seed region sequence, other members from the miR-130/301 family may also regulate BMPR2 expression. The transcription of miR-130a-3p is controlled by NF-κB in PAECs. Thus, NF-κB and BMPR2 are linked by miR-130a-3p. In addition, miR-17a-5p, miR-20a-5p, and miR-125a-5p also mediate BMPR2 expression. Besides, two members from the miR-17-92 family, miR-17a-5p and miR-20a-5p, link STAT3 to BMPR2 (Brock et al., 2009; Huber et al., 2015; Figure 8B).

In PAFs, activation of the miR-130/301 family can induce cell proliferation and extracellular matrix remodeling by inhibiting PPARγ and LRP8. Meanwhile, matrix remodeling can activate POU5F1, which subsequently promotes miR-130/301 family expression (Bertero et al., 2015). Thus, a positive feedback circuit is activated that dramatically accelerates the development of PH (Figure 8C).

**FIGURE 9:**
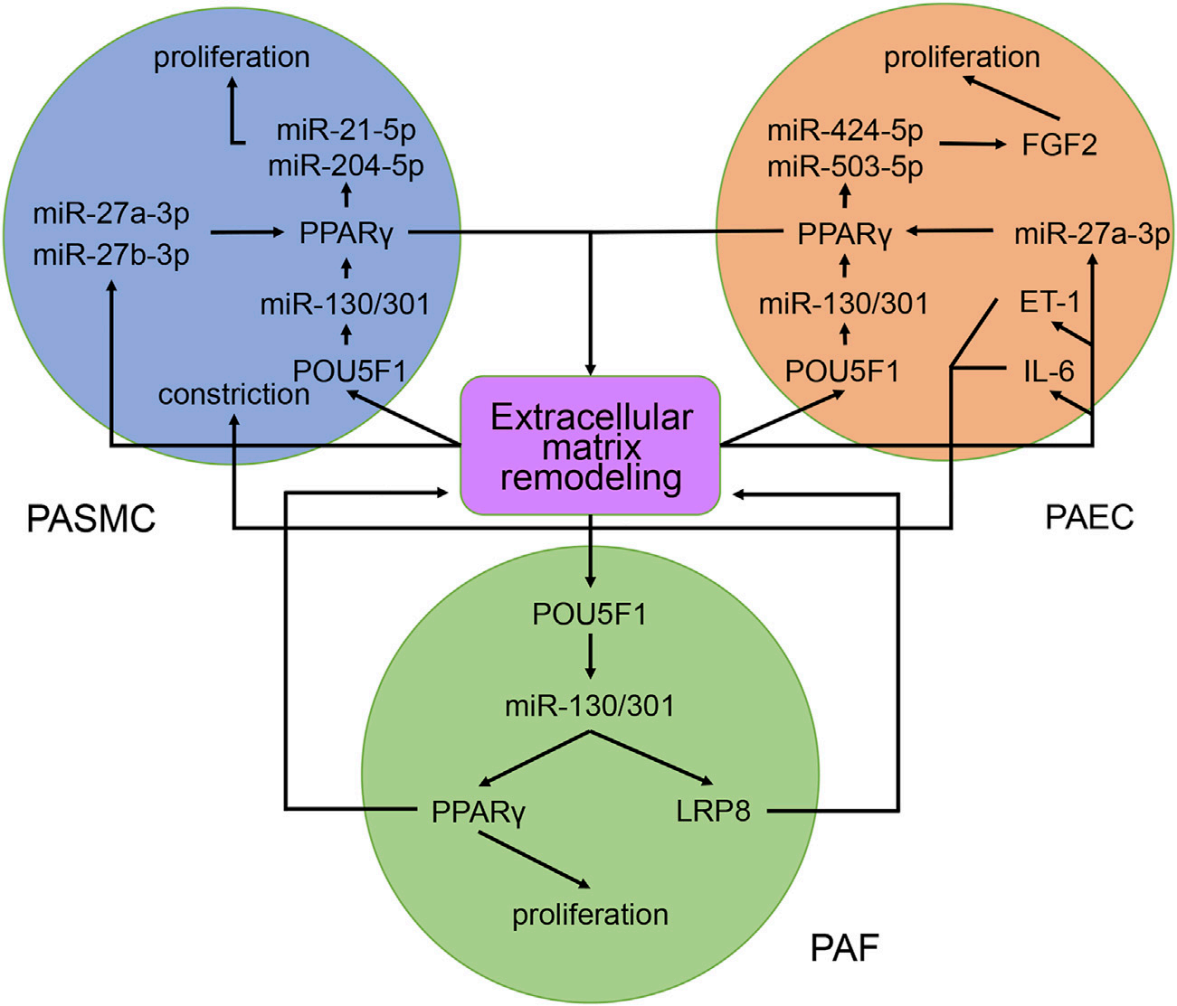
Contribution of the miR-130/301 family to the crosstalk between various pulmonary artery cells. The remodeled extracellular matrix induces proliferation and contraction in pulmonary artery cells via miR-130/301 family-dependent and -independent pathways. Meanwhile, matrix remodeling can be induced by overexpression of the miR-130/301 family.

The roles of the miR-130/301 family in different pulmonary artery cell types are not independent. Rather, the miR-130/301 family contributes to crosstalk between these cells. Extracellular matrix remodeling, which can be induced by overexpression of the miR-130/301 family, promotes proliferation and contraction of pulmonary artery cells viamiR-130/301 family-dependent and -independent pathways. The remodeled extracellular matrix can activate the POU5F1/miR-130/301 family/PPARγ axis in PASMCs, PAECs, and PAFs, subsequently regulating downstream molecules such as miR-204-5p, miR-424-5p, miR-503-5p, and FGF2 (Bertero et al., 2015). Upregulating miR-424-5p and miR-503-5p or inhibiting FGF2 in PAECs can repress PASMC and PAF proliferation induced by conditioned media from PAECs, indicating that these molecules are involved in the crosstalk among different pulmonary vascular cells. The remodeled extracellular matrix can also induce the expression of the proliferative miRNA, miR-27a/b-3p, in PACEs, and PASMCs, as well as the expression of the vasoconstrictor ET-1, and the inflammatory cytokine IL-6 in PACEs (Bertero et al., 2014; Bertero et al., 2015; Figure 9).

## 4 Environmental Factors in Pulmonary Hypertension

Noncoding RNA interference is an important epigenetic mechanism. Recent evidence has identified the roles of epigenetic changes in the development of PH. These changes link the pathogenic genes of PH and environmental factors such as hypoxia, virus infection, and air pollution (Gamen et al., 2016). For example, BMPR2 is a transmembrane serine/threonine kinase receptor, which is essential for vascular homeostasis. Although mutations in the BMPR2 gene account for a considerable portion of patients with familial pulmonary artery hypertension (FPAH), only 20–30% of carriers with mutations in this gene suffer from PH, indicating that other factors contribute to the onset of the disease (Orriols et al., 2017; Zhao et al., 2019). According to our network, hypoxia can induce the expression of several miRNAs by HIF-1α, such as, miR-145-5p, miR-19a-3p, miR-191-5p, miR-214-3p, and miR-27a-3p (Agrawal et al., 2014; Camps et al., 2014; el Azzouzi et al., 2013; Song et al., 2014; Zhao et al., 2019). Among them, miR-191-5p can increase cell proliferation, impair apoptosis, and induce phenotypic alteration through inhibiting BMPR2 expression, subsequently contributing to vascular remodeling (Song et al., 2014). Therefore, the HIF-1α/miR-191-5p/BMPR2 axis reveals the connection between hypoxia and BMPR2 expression and partially explains the incomplete penetrance of BMPR2 mutations in FPAH.

## 5 Potential Applications of Non-coding RNAs

Ultimately, studies on molecular mechanisms aim to inform clinical practices. NcRNAs are potential diagnostic biomarkers for PH. For example, circRNAs are not easily degraded, making them ideal serum biomarkers. Zhang et al. reported hsa_circ_0068481 overexpression in the serum from patients with IPAH. Furthermore, hsa_circ_0068481 expression is significantly correlated with 6-min walk distance, N-terminal pro-B-type natriuretic peptide, H2S, pulmonary hypertension risk stratification, right heart failure, and survival rate (Zhang et al., 2019a). However, because of the absence of an associated molecular mechanism, this circRNA was not included in our networks. NcRNAs may also act as potential therapeutic targets for PH. For example, Rothman et al. identified downregulation of miR-140-5p in a rat PH model. *In vitro*, miR-140-5p mimics suppressed PASMC proliferation and migration. *In vivo*, miR-140-5p mimics prevented the progression of established PH in rats (Rothman et al., 2016). The results are encouraging. However, ncRNA therapy is far from being applied in clinical settings, since a ncRNA may have diverse biofunctions. This means that when used as therapeutic agent, a ncRNA may cause adverse effects, some of which may even be life-threatening. In our opinion, carefully selected ncRNA targets and well-designed action sites can be helpful to avoid such adverse effects. These measures require a comprehensive and in-depth understanding of the mechanisms of ncRNAs in diseases. In this study, we constructed networks to demonstrate the current findings on ncRNAs from studies performed in PH patients and animal models. However, shortcomings of these studies, including the paucity of human data, sex bias, and heterogeneity of animal models, limit the translation of these findings into applications for human disease. Therefore, further studies should be performed to confirm these findings in different animal models and patient cohorts of PH. Additionally, large, well-designed, and unbiased clinical studies are required to illuminate further application of ncRNAs.

## 6 Conclusion

The roles of ncRNAs in PH remained unclear. In this study, we performed an extensive literature search and adopted uniform and strict criteria for the selection of each article to avoid biased outcomes. The ncRNA networks were constructed by assembling ncRNAs and their interacting RNAs or genes from included articles. These networks provide a better understanding of the roles of ncRNAs in PH and can be helpful in elucidating the potential clinical applications of ncRNAs.
